# The Basaglia Law. Returning dignity to psychiatric patients: the historical, political and social factors that led to the closure of psychiatric hospitals in Italy in 1978

**DOI:** 10.1177/0957154X231224650

**Published:** 2024-02-09

**Authors:** Valentina Badano

**Affiliations:** University of Aberdeen Medical School

**Keywords:** Asylums, Basaglia Law, Istituzione Negata, Italy, 1970s

## Abstract

Law no. 180 of 1978, which led to the closure of psychiatric hospitals in Italy, has often been erroneously associated with one man, Franco Basaglia, but the reality is much more complex. Not only were countless people involved in the movement that led to the approval of this law, but we should also take into account the historical, social, and political factors that came into play. The 1970s in Italy were a time of change and political ferment which made this psychiatric revolution possible there and nowhere else in the world.

## Introduction

Italy’s Law no. 180 (Legge del 13 maggio 1978, n. 180), known as the ‘Basaglia law’, is highly controversial and is argued to have been either a breakthrough in the field of psychiatry or an idealistic proposal that failed in practice (De Girolamo, Barale, Politi and Fusar-Poli, 2008). This law imposed the closure of Italian psychiatric hospitals in favour of a community-based approach and, more importantly, it returned civil rights to psychiatric patients (Camarlingi, 2008; Piccinelli, Politi and Barale, 2002). Many historical accounts only credit Franco Basaglia, the psychiatrist who initiated the anti-asylum movement in Italy, with the approval of this law (Stoppoloni, 2016). However, these explanations overly simplify the polycentric nature of a movement that involved many figures, inside and outside the medical field, as well as the patients themselves (Foot, 2015: Chapter 1, Section 2, Paragraph 43).^1^ As Basaglia often stressed, he could not have done this alone. The law was the result of a compromise between different psychiatric groups and political parties, and Basaglia was left dissatisfied with several aspects of it (Costanzo and Gazzara, 1979). Therefore, ‘the term ‘Basaglia law’ in itself . . . is in many ways a historical error’ (Foot, 2015: 23/6/1). Moreover, the movement would not have gained widespread support if it had not started at a time of social reforms and political ferment (Basaglia, Ongaro Basaglia, Pirella and Taverna, 1978/2008: 31). The aim of the present paper is to rediscover the often forgotten historical figures and events that advocated a change. This includes looking into the international, national, and local influences that helped to shape a new mental healthcare system in Italy, and the role of social movements and the media in mobilising public opinion.

## Psychiatric care in Italy before 1978: why was it a problem?

As stated above, Law no. 180 drastically altered the treatment of mental illness in Italy. The first law regulating Italian psychiatric care had been passed in 1904 (Legge del 14 Febbraio 1904, n. 36) when the conditions inside asylums were atrocious; there was overcrowding, poor hygiene, scarcity of food, and repeated abuse of patients. An inquiry conducted in 1901 publicised this situation and a scandal arose causing political action (Babini, 2009: 9, 13, 17–18; Piccinelli et al., 2002).

However, the objective of the 1904 law was not to improve the treatment of patients but to remove them from society due to their perceived dangerousness and indecency and thus to avoid public scandal. Citizens could report others, leading to forced institutionalisation, but people in need could not themselves demand treatment. The directors of the asylums were in charge of admissions and discharges, so they had control over the lives of patients (Babini, 2009: 19–20). When patients were admitted, they would lose the right to vote and become excluded from society (Zinnari, 2021). Furthermore, they were not involved in decisions about the care provided for them, and this resulted in forced treatments. Scandals and reports on the matter continued, but these were generally ignored as the wider public had no interest in advocating for the mentally ill, who were detained in asylums away from everyone’s sight (Babini, 2009: 18–20; Zinnari, 2021).

There was a minor improvement in 1968 when Legge del 18 marzo 1968, n. 431 introduced the option of self-referral to asylums, but Law no. 36 remained the ruling law on psychiatric care until 1978. According to Basaglia, this law caused ‘the sick [to enter] the hospital as a man, after some time they would become a thing. A thing mortified and violated by the hospital, by the institution, by the rules of the institution’^2^ (Pierelli and Pozzi, Season 2017/2018). As stated by a patient in the asylum in Gorizia, the patients’ only escape from asylums was, more often than not, in a coffin (Basaglia, 1968/2013: 14). In the 1970s, the cruel conditions in asylums caused politicians such as Mario Tommasini, assessor for the Province of Parma, and psychiatrists such as Agostino Pirella, to compare asylums to concentration camps, thus sparking political debate (Foot, 2015: 5/4/1; 18/1/6). Clearly, a change was needed.

## The role of international systems in reshaping Italian psychiatric care

Considering the bias of Italian psychiatric culture towards a traditional approach, influence from other countries was pivotal in implementing change (Orsini, 2019). This happened in Italy in the 1960s, a decade later than in other Western countries (Micheli, 2019). Two main factors led to innovations: firstly, the pharmacological breakthrough in 1950s France, which enabled medical management of acute psychiatric attacks and opened possibilities for new treatments (Babini, 2009: 161); secondly, the increased accessibility of travel after World War II allowed specialists to observe new models of care in the USA, the UK, and France. Psychiatrists in Italy imported the American idea of replacing asylums with community mental health centres (Micheli, 2019). This model was similar to the work of the psychiatrist Giovanni Jervis in Reggio Emilia and Tommasini in Parma (Foot, 2015: 18/4/2; 19/1/9). Following deinstitutionalisation, the mortality rates in the USA increased. However, in Italy, this did not happen, probably because of the public’s support for the change – support which was lacking in the USA (Dumont and Dumont, 2008).

The ‘therapeutic community’ model from the UK was followed in Gorizia by Basaglia and his team, who were the first reformers. It involved maintaining the old asylum, but introducing democracy by modernising the rules and giving more freedom to patients. The renowned psychiatrist Maxwell Jones implemented this method, and introduced Basaglia to it (Foot, 2015: 7/10; Micheli, 2019). Jones’ experiment involved discussions between doctors and patients about treatment and allowed inpatients to have short trips outside the asylum (Micheli, 2019). Basaglia partially adapted this, as patients’ and doctors’ discussions focused mainly on practical and social issues instead of treatment. He also introduced general assemblies where everyone could participate. Both these innovations empowered patients and built a sense of community (Foot, 2015: 7/1/1,2; 7/2/1–5; 10/1).

The ‘psychiatric sector’ and the ‘*psychothérapie institutionelle*’ were imported from France. The former aimed to turn an area of the city into the centre of care, instead of the asylum, which allowed for the reintegration of patients into society (Micheli, 2019). This idea, alongside the model of the ‘Therapeutic Community’, inspired Basaglia’s work in Trieste and proved to be successful (Foot, 2015: 22/2/1). The latter concept aimed to improve patients’ conditions by employing psychoanalytical psychotherapy inside asylums. New training for staff was necessary in order to achieve this. Unfortunately, this model was not always used correctly; for example, it failed in Varese because only new staff were trained in the new methods and this caused conflicts with existing personnel (Micheli, 2019).

## The local influences: a variegated picture

Several Italian cities tailored these international models to their own needs, modernising psychiatric treatment while challenging the role of psychiatrists (Basaglia, 1973/1985; Becker and Fangerau, 2018). Basaglia revolutionised Gorizia’s asylum in 1961 and then Trieste’s in the 1970s; the changes he made in Trieste, and a press conference he held there in 1977, helped to promote the ideas which made it possible for Law no. 180 to be approved in 1978 (Ongaro Basaglia, 1998). Similar ideas had also spread to other cities such as Perugia, Parma, Arezzo, and Reggio Emilia. Different approaches were implemented, but with a shared goal: give freedom and dignity back to patients, close asylums, and provide alternative treatments (Foot, 2015: 19/1/12–13). Finally, patients were no longer considered dangerous or incurable (Bongiorno, 2013).

The politics of each city deeply affected the development of different strategies. Gorizia, for example, was dominated by centre-right politics with a strong presence of the Neo-Fascist Party. This caused opposition to the opening of decentralised services, so changes were only implemented inside the asylum (Foot, 2015: 1/2/31; 20/1/4). This included banning cruel treatments such as shock treatments, opening wards, and setting up services and workspaces for patients (Burti, 2001; Foot, 2015: 7/7/1).

In other reformist cities, the parties of the left were in charge, allowing for the opening of mental health centres, emptying of asylums, and reintegration of patients into society. Politicians such as Ilvano Rasimelli and psychiatrists like Carlo Manuali promoted such changes in Perugia with the support of the Communist Party, one of the strongest leftist parties at the time. Communist support was pivotal, as in other cities Communists had more conservative approaches towards change, which led to unfavourable outcomes. Some branches supported the reforms while others preferred big institutions as they facilitated the spread of Communism. The latter situation occurred in Trieste, complicating the work of Basaglia, and in Parma, where Tommasini operated. Political endorsement was crucial for centres to be opened and function as alternatives for asylums (Babini, 2009: 264; De Girolamo, Bassi, Neri et al., 2007; Foot, 2015: 18/9/1; 17/2/1–2; 17/3/1; 19/2/16; 22/6/1).

The promoters of these innovations also differed among cities. In Gorizia, the hospital staff promoted change through a revolutionary and contradictory stance: they worked inside the asylum, aiming to destroy it (Basaglia, 1968/2013: 11). In contrast, in Parma the staff opposed the closure of the asylum, a major source of wealth in the city. There, the reformers did not have a medical background, but were political figures, volunteers, and students (Foot, 2015: 18/7/5; 18/11/4.). Similarly, in other cities the public also got involved. This happened due to links between the social movement that invested Italy in the 1960s and the anti-asylum movement.

## The negated institution: a connection between the social reforms of 1968 and the anti-asylum movement

In 1968, student protests started in Italy before spreading worldwide. They promoted anti-authoritarianism and direct democracy; they also opposed institutions and capitalism (Hilwig, 2009: 3, 10, 16, 24). In Italy, students joined the protests of factory workers, culminating in several reforms that improved individual rights such as the liberalisation of university access and the divorce law (Becker and Fangerau, 2018; Hilwig, 2009: 5).

Connecting the anti-asylum movement with social change was the book *L’Istituzione negata* (The Negated Institution), first published in 1968 (Foot, 2015: 10/3/8). It contained pieces written by Basaglia, members of his team, the journalist Nino Vascon, and transcripts of patient interviews and meetings which helped in vocalising patients’ experiences (Basaglia, 1968/2013: 11–14). Surprisingly, the 1968 book became a bestseller and a crucial text for the student movement (Babini, 2009: 246). Einaudi, one of the main printing businesses at the time, published it owing to favourable ties with the Gorizian team. Einaudi largely promoted the book through an advertising campaign, allowing it to reach a wide audience which helped in publicising the anti-asylum ideas (Foot, 2015: 11/1/1, 21).

The reasons for the success of the book were several. It criticised capitalism and all institutions, while providing a realistic plan using Gorizia as an example. It stated that collective effort was the foundation for change, while also advocating the rights of the marginalised (Babini, 2009: 244; Basaglia, 1968/2013: 4–7). This agreed with the ideals of the protestors, making it extremely popular. These links between the student and psychiatric movement decreased stigma towards mental illness and encouraged unification towards a common goal (Babini, 2009: 188).

The success of the book made Gorizia’s asylum famous, as well as Basaglia and his team, allowing them to move to different cities and work there spreading their ideology further. Moreover, asylums started to be used as assembly centres for the 1968 social movement. Intellectuals, photographers, and others became interested in asylums, and documentation of the struggle started. As interest grew, support increased, and Basaglia finally obtained political support locally and nationally (Foot, 2015: 13/4/4; 11/1/26; 13/4/4).

## The impact of the media in mobilising public opinion

Changing the public’s attitude towards mental illness was not just due to *L’Istituzione negata*. All media played a key role in influencing the public’s perception of asylums. Documentaries about them, such as ‘The Snake Pit’, started circulating, but only among specialists. This was because the Italian psychiatric academic world feared a backlash if people discovered the patients’ horrible conditions in asylums. One particularly revolutionary documentary was Sergio Zavoli’s ‘*I giardini di Abele*’ (The Gardens of Abel) filmed in 1968 in Gorizia’s asylum. It was broadcast in the evenings of the Christmas holidays in 1969, thus guaranteeing a wide public reach. For the first time, Italians were shown the conditions inside asylums, causing outrage. The aim of the documentary was to shake people’s moral conscience and portray the asylum problem as a wider societal issue (Babini, 2009: 7– 8, 137–8). Following other media, it also drew parallels between psychiatric hospitals and concentration camps. Additionally, it investigated how the patients’ social class influenced the level of care they received (Foot, 2015: 15/1/5-6, 11). Zavoli’s documentary paved the way for other important depictions of life inside asylums, like *Matti da slegare* (‘Fit to be Untied’) filmed in Parma in 1975 (Babini, 2009: 266).

Other key components that influenced public opinion were the book *Morire di classe* by photographers Gianni Berengo Gardin and Carla Cerati (1969) and a statue made by the artist Giulano Scabia. This book, which was released in the same year as the film *I Giardini di Abele*, publicised asylum closure through shocking visuals. Pictures were taken in Gorizia, Parma, and Florence, all reformed asylums, as if they were still traditional institutions, in order to shock the population (Foot, 2015: 15/2). Another important initiative was the construction by Giuliano Scabia of a blue horse called Marco Cavallo for Trieste’s asylum. The statue contained papers with the patients’ dreams written on them and it was paraded around the city followed by a group of patients to show that they were individuals worthy of rights and freedom (De Marinis and Vannucci, 1977).

Journalists, such as Angelo di Boca and Nino Vascon, also played a key role in spreading the ideas of the movement nationally with their articles (Foot, 2015: 6/1/2; 11/1/24). Creative ideas involving newspapers also began circulating; for example, in the asylum of Gorizia patients and staff could write articles in *Il Picchio*, aiming to create a group of leaders among the patients to help in the fight (Basaglia, 1968/2013: 82–3). The local press, however, often opposed the reintegration of mentally ill people into the community, sometimes leading to conflicts between reformers and locals in cities with asylums. Furthermore, journalism at the time mainly followed Basaglia, so other initiatives were not publicised and are now forgotten (Foot, 2015: 18/6/6; 20/1/3; 17/9/7).

## The mobilisation of politicians and the approval of the law

After years of work to publicise the struggle, the anti-asylum battle started to gain support from left-wing parties and the public (Burti, 2001). In 1977, discussions started in Parliament regarding the National Health Reform (Legge del 23 dicembre 1978, n. 833) to make healthcare public. This included debates over a possible reform of the asylum system, despite the actual implementation of the law being unlikely (Babini, 2009: 286–8). Then, unexpectedly, the Radical Party collected enough signatures for a referendum for the abolition of Law no. 36 of 1904. The referendum would have gone ahead if another law to replace Law No. 36 was not passed before it (Burti, 2001). It could seem that if the referendum succeeded and Law no. 36 was abolished, this would be in line with the ideas of the anti-asylum movement while also bringing even more attention to the cause. However, whatever the result of the referendum might have been, it posed issues. If the law was abolished, this would have left psychiatric hospitals without any legal structure until the National Health Reform was approved, causing chaos; if the population voted to keep the traditional asylums, then future change in legislation would prove difficult since a major part of the population favoured the current situation (Babini, 2009: 288; Foot, 2015: 23/4/3–4).

Therefore, an emergency procedure was implemented in 1978 allowing discussion and approval of the law by health commissions only, thus bypassing parliamentary debate. The leading parties, the Communists and Christian Democrats, worked together and the new legislation was approved in less than a month (Becker and Fangerau, 2018; Foot, 2015: 23/4/5–6). Alongside politicians, the differing psychiatric groups also worked together to write the law (Costanzo and Gazzara, 1979). Therefore, it would be wrong to attribute it to Basaglia alone, especially as the main promoter of the law in Parliament was Bruno Orsini, a psychiatrist and Christian Democrat, whose views differed from Basaglia’s. Arguably, the law should have been named after him (Babini, 2009: 288).

Due to this urgency, the infrastructures needed for the innovations were not yet established, so the practical impact of the law was minimal (Costanzo and Gazzara, 1979; Ongaro Basaglia, 1998). The law allowed regions to manage independently the allocated funds for psychiatric care, resulting in variations in the level of treatment provided (Ferranini and Peloso, 2016). In the following years, this, and other issues, generated continuous problems which were often attributed to Basaglia, despite the fact that several other figures also contributed to the law. They all knew its limitations, but time was of the essence (Camarlingi, 2008; Costanzo and Gazzara, 1979). This legislation’s real achievement was the guarantee of patients’ rights in line with the Constitution, and the establishment of their equality with any other Italian citizen (Orsini, 2019). Law no. 180 also prohibited the admission of new patients to asylums and the construction of new psychiatric hospitals, which was a victory for the anti-asylum movement (Legge del 13 Maggio 1978, n. 180). In reality, the closure of all asylums took years, and Law no. 180 was subjected to continuous attacks. However, it still stands now absorbed in the National Health Reform, granting freedom to psychiatric patients.

## Conclusion

Franco Basaglia was involved in all aspects of the process that returned rights to psychiatric patients. However, he was not the only one who fought in a period where change was felt in every part of Italian society. Before 1978, psychiatric hospitals were places where patients were segregated to remove their unwanted presence from society, rather than to provide care. Almost no improvements were made before Law no. 180, as the Italian psychiatric scene rejected any new approaches. Change started happening only in the 1960s with the introduction of psychiatric medications and international models of treatment. The political situation in different areas also influenced the type of care provided, while the economic weight of the asylum had a role to play in who promoted the innovations. The protagonists came from a variety of backgrounds, not just medical, with students playing a prominent role. The connection of the students’ and workers’ movements with the psychiatric one caused the wider public to become interested in the anti-asylum struggle. This happened primarily due to the success of the book *L’Istituzione negata* and the efforts of different media which publicised the atrocious situations inside psychiatric hospitals. Not all news outlets were supportive, but the notion that change was essential still reached the public, resulting in political support and the approval of Law no. 180. The law was passed quickly due to the fear of a referendum, but this is not to undermine its importance. The new legislation was not perfect, but patients were finally considered citizens again. In the words of Franca Ongaro Basaglia (Basaglia’s wife and a key member of his team), ‘there were people who refused to accept the fact that there in a hospital the patients could be destroyed, annihilated, crushed . . . and those people started to do something’ (Foot, 2015: 24/1/4).

## Notes

1. An online version of this book (which has no page numbers) was used, so the citations give the chapter, section and (if available) paragraph numbers; all further citations for the book will be in the format: (Foot, 2015: 1/2/43)

2. Translated from Italian by the present author.
